# The Maxillary Nerve Block in Cleft Palate Care: A Review of the Literature and Expert’s Opinion on the Preferred Technique of Administration

**DOI:** 10.1097/SCS.0000000000010343

**Published:** 2024-06-11

**Authors:** Jess J. Peters, Karl Jacobs, Montserrat Munill, Anke P.C. Top, Markus F. Stevens, Elsa M. Ronde, J. Peter W. Don Griot, Nadia Lachkar, Corstiaan C. Breugem

**Affiliations:** Departments of * Plastic, Reconstructive and Hand Surgery, Amsterdam UMC, location University of Amsterdam, Amsterdam, the Netherlands; § Medical Biology, Section Clinical Anatomy and Embryology, Amsterdam UMC, location University of Amsterdam, Amsterdam, the Netherlands; ¶ Anaesthesiology, Amsterdam UMC, location University of Amsterdam; † Amsterdam Reproduction and Development Research Institute, Amsterdam, The Netherlands; ‡ Oral Pain and Dysfunction, Functional Anatomy, Academic Centre for Dentistry Amsterdam (ACTA), University of Amsterdam and VU University Amsterdam, Amsterdam, The Netherlands; ∥ Oral and Maxillofacial Surgery, University Hospital Vall d’Hebron, Barcelona, Spain

**Keywords:** Cleft palate, maxillary nerve block, pain management

## Abstract

**Introduction:**

Although the maxillary nerve block (MNB) provides adequate pain relief in cleft palate surgery, it is not routinely used globally, and reported techniques are heterogeneous. This study aims to describe relevant anatomy and to present the preferred technique of MNB administration based on the current literature and the expert opinion of the authors.

**Method and materials:**

First, a survey was sent to 432 registrants of the International Cleft Palate Master Course Amsterdam 2023. Second, MEDLINE (PubMed interface) was searched for relevant literature on maxillary artery (MA) anatomy and MNB administration in pediatric patients.

**Results:**

Survey response rate was 18% (n=78). Thirty-five respondents (44.9%) used MNB for cleft palate surgery before the course. A suprazygomatic approach with needle reorientation towards the ipsilateral commissure before incision was most frequently reported, mostly without the use of ultrasound. Ten and 20 articles were included on, respectively, MA anatomy and MNB administration. A 47.5% to 69.4% of the MA’s run superficial to the lateral pterygoid muscle and 32% to 52.5% medially. The most frequently described technique for MNB administration is the suprazygomatic approach. Reorientation of the needle towards the anterior aspect of the contralateral tragus appears optimal. Needle reorientation angles do not have to be adjusted for age, unlike needle depth. The preferred anesthetics are either ropivacaine or (levo)bupivacaine, with dexmedetomidine as an adjuvant.

**Conclusion:**

Described MNB techniques are heterogeneous throughout the literature and among survey respondents and not routinely used. Further research is required comparing different techniques regarding efficacy and safety.

Cleft lip and/or palate is a common congenital malformation with a worldwide prevalence of ∼1:700 births.^1^ Children suffering from cleft lip and/or palate often require multiple surgeries since cleft palate repair is essential for proper feeding and speech development.^2–4^

Despite being a challenge, especially in young children, postoperative pain relief is of great importance since insufficient pain management hinders the initiation of oral food intake and could result in a longer hospital stay.^5^ Since cleft surgeries are generally very painful procedures,^2,3,6^ morphine is routinely used in the Amsterdam UMC after cleft palate repair in pediatric patients. Nevertheless, reasons to strive to limit morphine use in young children are postoperative sedation, nausea, vomiting, and respiratory depression,^6–9^ to which infants are more sensitive compared to older children or adults.^8^

Literature has shown the maxillary nerve block (MNB) to be an effective addition to pain management in children undergoing cleft palate repair.^10^ This peripheral nerve block achieves direct analgesia of both the hard and soft palate and is reported to decrease postoperative pain scores,^4^ postoperative rescue analgesic consumption,^2,4,5,9,11,12^ as well as time to oral feeding.^4,5,11^ Various techniques have been described for MNB administration in the literature, including intraoral approaches used mainly in dentistry^13–17^ and infrazygomatic approaches for the treatment of trigeminal neuralgia in adults.^18,19^ Different suprazygomatic approaches have been described in relation to cleft palate surgery in pediatric populations.^2,4–6,11,20–23^ However, the described techniques remain heterogeneous, and the MNB is not yet routinely used globally in cleft palate care.

The authors of this article organized an international hybrid course on local nerve blocks in cleft palate surgery to bring international expert speakers together to explore MNB administration techniques. All registered participants were invited to share their experience in cleft palate surgery-related MNB use through a survey after the course. The purpose of this study is to present the results of the survey describing course participants’ preferred MNB administration techniques in cleft palate surgery, as well as provide a narrative review of the current literature on maxillary artery (MA) position in relation to the lateral pterygoid muscle (LPM), MNB administration techniques, commonly used anesthetics and adjuvants, and the added value of ultrasound guidance.

## MATERIALS AND METHOD

### Survey Methodology

A survey was sent to 432 registrants of the International Cleft Palate Master Course Amsterdam (ICPMCA) 2023. The Medical Ethics Review Committee of the Amsterdam UMC reviewed this study and exempted it from official approval (2023.0204).

The ICPMCA23 was held on MNB administration techniques in cleft palate surgery in February 2023. The course was promoted through Stichting Interplast Holland and Smile Train, and the course equipment was facilitated by Rods and Cones, Mindray, and GE Healthcare. Of the total 432 registrations, 13 participants attended the course live. The course consisted of multiple presentations by expert speakers and a hands-on anatomic wet laboratory session during which different MNB administration techniques were performed with and without ultrasound guidance on “Fix for Life”^24^ conserved adult cadaveric heads. Different colored pastes were used for the different administration techniques to determine the location of the injectate during dissection. All the presentations and a live-streamed block administration with colored paste and dissection were available for the online attendees.

In April 2023, an online cross-sectional survey was sent to all ICPMCA23 registrants using Castor Electronic Data Capture.^25^ A reminder was sent 2 weeks later. The survey questions were developed by the authors and consisted of a maximum of 50 questions, including questions on the respondent’s use of MNB in cleft palate surgery before the course (Supplemental Appendix A, Supplemental Digital Content 1, http://links.lww.com/SCS/G343). All answers were collected anonymously.

Quantitative survey data were analyzed using SPSS (v28.0.1.1; IBM Corp, Armonk, NY),^26^ and are presented as numbers (N) and proportions (%) of survey respondents.

### Literature Search Methodology

A literature search was conducted in MEDLINE (PubMed interface) for relevant literature on anatomic variations of the MA by combining the terms “maxillary artery” and “anatomy.” The filters “human,” “English” and 2010 to 2023 were applied. A second literature search was conducted using the same source for relevant literature concerning MNB administration in pediatric populations by combining the terms “maxillary nerve,” “block” and “children” or “infants.” Records in English describing the course of the MA or the use of MNB in pediatric populations were included. Case reports, records in another language, records where full texts could not be retrieved, and records not describing, respectively, the course of the MA or MNB administration were excluded. All articles included on the subject of MNB administration were critically appraised using the critical appraisal tools from the Joanna Bricks Institute. Depending on the type of article, the checklist for randomized controlled trials,^27^ analytical cross-sectional studies,^28^ cohort studies,^28^ or textual evidence: expert opinion^29^ was used. Data on the course of the MA in relation to the LPM or MNB administration techniques, as well as anesthetics and adjuvants in pediatric populations, were extracted from the included records. The results of both searches are presented in a narrative synthesis.

## RESULTS

### Survey

The survey aimed to identify the current use of MNB in cleft palate surgery among international professionals that registered for the course. A total of 78 registrants (18% response rate) from 37 different countries responded to our questionnaire. Supplemental Table 1, Supplemental Digital Content 2, http://links.lww.com/SCS/G344 overviews survey respondent characteristics.

**FIGURE 1:**
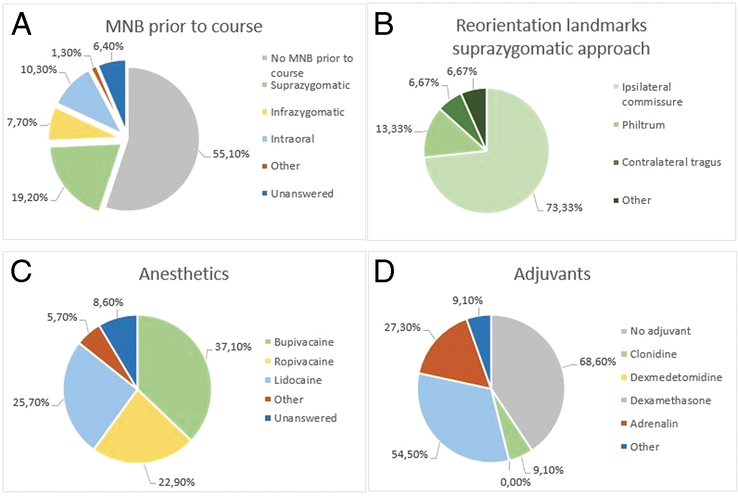
(A) Reported MNB use before the course and used techniques of administration. (B) Landmark to which the needle is directed when using a suprazygomatic approach for MNB administration before the course. (C) Type of local anesthetics used by respondents that used MNB before the course. (D) Type of adjuvants used by respondents that used MNB before the course. MNB indicates maxillary nerve block.

Thirty-five (44.9%) respondents reported using MNB for cleft palate surgery before the course. With regard to the technique of administration, a suprazygomatic approach with needle reorientation towards the ipsilateral commissure was most frequently reported (Fig. 1A and B). The most frequently used anesthetic for the MNB was bupivacaine, followed by lidocaine and ropivacaine (Fig. 1C). Several different concentrations and injected volumes of anesthetics were reported, ranging from 0.25% to 0.5% for bupivacaine with a maximum volume of 1 to 3 ml per side, 0.2% to 3% for ropivacaine with injected volumes of 1 to 5 ml and 1% to 2% for lidocaine with total injected volumes of 2 to 6 ml. Eleven (31.4%) respondents usually added an adjuvant to the block. The most commonly reported adjuvant was dexamethasone 1 to 4 mg, followed by adrenalin 1:100,000 to 1:200,000 and clonidine 1 µg/kg (Fig. 1D). Adrenaline and clonidine were always administered within the block, whereas 67% of dexamethasone was administered intravenous (IV) and the remaining 33% within the block. Seven (20%) respondents who performed the MNB before the course used ultrasound-guided administration. Three respondents from Vietnam, Tanzania, and Colombia used ultrasound guidance whenever available (8.6%). Administration of the MNB before incision was reported by 28 (80%) respondents, and only 2 (5.7%) reported administering the MNB postoperatively. For the remaining 5 (14.3%) respondents, the moment of MNB administration was unanswered. The use of local submucosal infiltration of anesthetics in addition to the MNB was reported by 21 (60%) respondents.

During dissection of the cadaveric heads after colored paste injection using different MNB administration techniques at the ICPMCA23, the paste was best located near the entrance of the pterygopalatine fossa (PPF) using an ultrasound-guided suprazygomatic approach with the contralateral tragus as landmark for needle reorientation. During the suprazygomatic approach, the needle was visible out of plane on the ultrasound image. Two bony landmarks were visible on the ultrasound image: the maxilla and coronoid process. It was observed that the second landmark moved when opening the mouth and was therefore considered to be the coronoid process of the mandible.

### Literature

A total of 233 articles on anatomic variations of the MA were screened for inclusion, and 7 articles were included. Through reference screening 3 additional articles were identified (see Supplemental Appendix B, Supplemental Digital Content 3, http://links.lww.com/SCS/G345 for the flowchart of study inclusion). The second search on MNB administration in children yielded 54 records, of which 13 were included. One additional article was included through reference screening. Six more articles on MNB techniques in adult populations were identified through reference screening and incorporated in this narrative review (see Supplemental Appendix C, Supplemental Digital Content 4, http://links.lww.com/SCS/G346 for the flowchart of study inclusion). The critical appraisal of all included articles on MNB administration is presented in Supplemental Appendix D, Supplemental Digital Content 5, http://links.lww.com/SCS/G347. Supplemental Appendix E, Supplemental Digital Content 6, http://links.lww.com/SCS/G348 provides an overview of study characteristics and extracted data from all included studies.

### Anatomy

**FIGURE 2:**
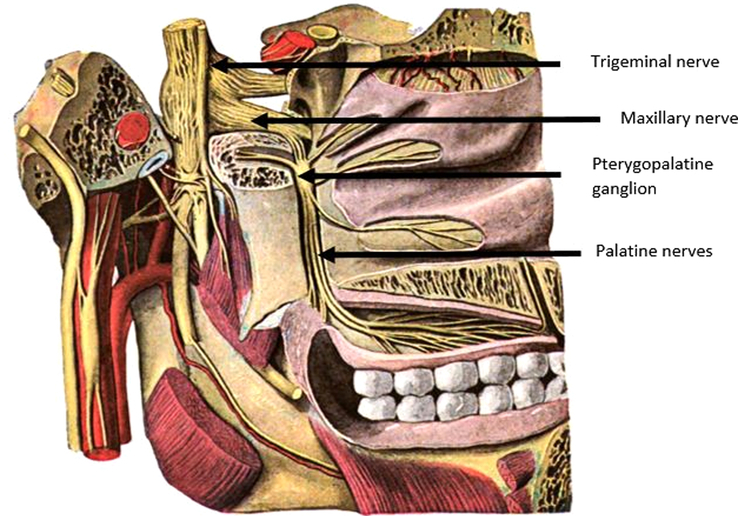
The palatine nerves arise from the pterygopalatine ganglion of the maxillary nerve within the pterygopalatine fossa (PPF). The maxillary nerve is the second branch of the trigeminal nerve.^31^

**FIGURE 3:**
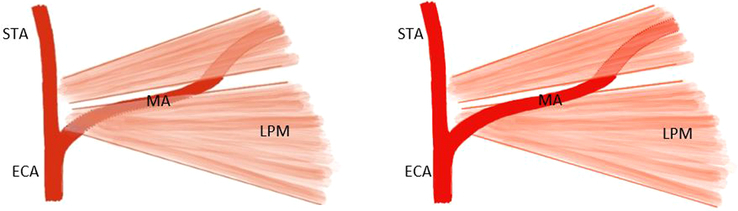
Deep (left) and superficial (right) course of maxillary artery (MA) in relation to the lower head of the lateral pterygoid muscle (LPM). ECA indicates external carotid artery; MA, maxillary artery; STA, superior temporal artery (based on an illustration by Uysal et al^40^).

The innervation of both the hard and soft palate is provided by branches of the maxillary nerve, which is the second branch of the trigeminal nerve (cranial nerve V) and contains only sensory fibers^30^ (Fig. 2). When aiming for the pterygopalatine ganglion positioned within the PPF to provide anesthesia of the palate, it is important to realize the needle will pass through the infratemporal fossa. The infratemporal fossa is an irregularly shaped cavity containing multiple structures, including the MA, branches of the maxillary and mandibular nerves, the pterygoid muscles, and the pterygoid venous plexus.^32^ To avoid complications such as damage to the MA or intra-arterial injection, awareness of the anatomic variations of the MA within the infratemporal fossa is of great importance, particularly in relation to the lower head of the LPM. In most cases (47.5%–69.4%), the artery has a lateral course,^33–40^ and in 32% to 52,5%, it runs medially to the LPM^33–36,38–40^ (Fig. 3). Asian populations are reported to have a higher incidence of the superficial variant of the MA, occurring in 82% to 97%.^33,34,36,37,40–42^ A third variant is reported in the literature to occur in ∼0.5% to 7% of patients.^36,37,39,41^ In these cases, the MA has neither a medial nor a lateral course but pierces the lower head of the LPM.^36,37,39,41^ Furthermore, an asymmetric course of the MA within the infratemporal fossa might occur in up to 21% of all cases.^36–39,42^

### Technique of Maxillary Nerve Block Administration

The maxillary nerve can be reached within the PPF via an intraoral approach by passing the needle through the greater palatine canal.^13–15^ To determine the location of the greater palatine foramen, the second and third molars need to be identified.^13,14^ Another intraoral approach often used in maxillofacial surgery and dentistry is the posterior superior alveolar nerve block, which mainly provides anesthesia of the ipsilateral molars.^16,17^ The needle is placed intraorally at the buccal side between the second and third molars and moved upwards towards the maxillary tuberosity and over the maxillary sinus towards the entrance of the posterior superior alveolar nerve into the bony canal of the maxillary sinus.^17^

When using an infrazygomatic approach, the needle is inserted inferiorly to the zygomatic arch and advanced towards the PPF,^18,19^ either in an anterior-to-posterior or posterior-to-anterior direction.^43^ Singh et al^18^ noticed that the needle could enter the orbit through the inferior orbital fissure in dry skulls if advanced beyond the pterygoid plate during a posterior-to-anterior infrazygomatic approach. Due to this possible complication, Stechison and Brogan^19^ used computed tomography (CT) imaging to confirm needle positioning when using an infrazygomatic approach to the maxillary nerve in adult patients with atypical posttraumatic facial pain.

**FIGURE 4:**
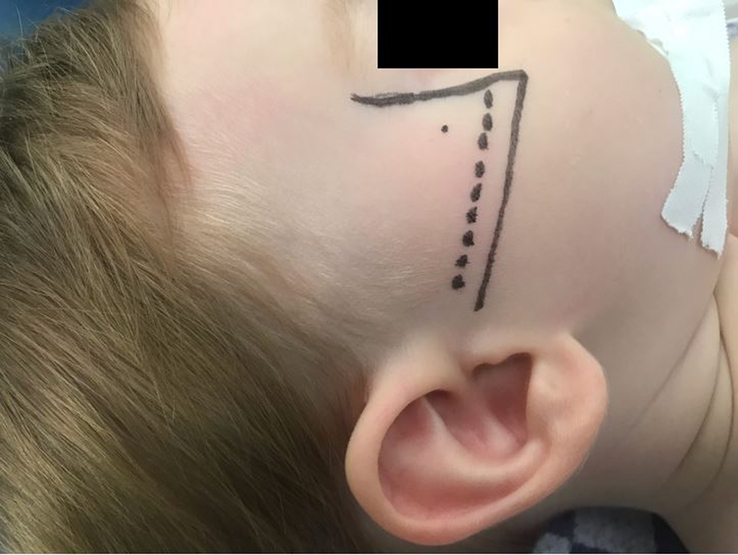
Placement of the needle perpendicular to the skin at the frontozygomatic angle formed by the lateral orbital rim and the zygomatic arch when performing a SZMNB (based on figure by Chiono et al.^2^) SZMNB indicates suprazygomatic maxillary nerve block.

In clinical studies on the use of MNB in cleft palate surgery in pediatric populations, the most frequently described technique is the suprazygomatic approach.^2,4–6,11,20–23^ However, there is some variation in the described administration techniques of a suprazygomatic maxillary nerve block (SZMNB). A few anatomic studies have described the preferred needle insertion point and reorientation angles to place the anesthetic near the pterygopalatine ganglion.^44–47^ The studies of Mireault et al,^44^ Prigge et al,^46^ and Captier et al^47^ agreed on inserting the needle perpendicular to the skin at the angle formed by the superior edge of the zygomatic arch and the lateral orbital rim (frontozygomatic angle) until it reaches the greater wing of the sphenoid in the temporal fossa (Fig. 4). With regard to the reorientation landmark, Mireault et al^44^, Captier et al,^47^ and Marston et al^45^ suggested reorienting the needle towards the philtrum to reach the PPF, while Prigge et al^46^ suggested reorientation of the needle towards the anterior aspect of the contralateral tragus based on measurements on dried pediatric skulls and dissected pediatric cadavers. Echaniz Barbero et al^48^ used the reorientation angles described by Prigge et al^46^ and found the methylene blue dye to be placed within the PPF and not to have spread into the orbit or intracranial fossa. Contrastingly, Mireault et al^44^ observed that the methylene blue dye had been injected within the infratemporal fossa and not directly within the PPF using the suprazygomatic approach with reorientation towards the philtrum.

A 24 to 27 G 38 to 50 mm needle is commonly used for SZMNB administration.^2,4–6,11,22,23^ The reorientation angles do not differ significantly between different age groups and, therefore, will not have to be adjusted for age.^45,47^ The depth of the needle advancement, however, will have to be adapted for age, varying from 20 mm in neonates^46^ to 30 to 38.55 mm at 0 to 1 years,^45,46^ and up to 47.11 mm at 18 years.^45^ In all clinical studies, the MNB is performed before incision,^2,4–6,11,20–22^ and some studies also describe local submucosal infiltration of either epinephrine^2,11,22^ or both epinephrine and lidocaine^5,23^ before incision, usually to improve hemostasis.

### Ultrasound Guidance

In the case of ultrasound-guided SZMNB administration, the transducer is usually positioned over the maxilla caudal to the zygomatic arch at a 45-degree angle in both horizontal and frontal planes,^4,5,22,44^ allowing visualization of the infratemporal fossa and MA.

### Local Anesthetics and Adjuvants

Many clinical studies performing MNB in children use either ropivacaine or levobupivacaine.^2,5,11,20–22^ Frequently used concentrations in clinical studies are 0.15 ml/kg of 0.2% to 0.25% for ropivacaine^2,5,11,22^ and 0.15 to 0.2 ml/kg of 0.125% or 0.25% for (levo)bupivacaine^4,6,20,23^ per side. A maximum total injected volume of 4^6,20^ to 5 ml^23^ per side is sometimes mentioned.

Adjuvants named in combination with an MNB in pediatric populations include dexamethasone IV,^21^ clonidine,^23^ and dexmedetomidine.^6^

Dexamethasone is an anti-inflammatory glucocorticosteroid that can be used as an adjunct within peripheral nerve blocks^49,50^ or IV.^21,50^ Esfahanian et al^21^ compared children undergoing cleft palate repair that received an SZMNB with ropivacaine to a group that received additional intraoperative and postoperative dexamethasone IV. They found the intervention group to have a statistically significant reduction in intraoperative and postoperative opioid consumption and a reduction in time to first oral intake.^21^

With regard to clonidine, Echaniz Barbero et al^23^ compared the effect of an SZMNB with bupivacaine alone to a combination of bupivacaine and clonidine in children undergoing cleft palate surgery. They found a significantly decreased intraoperative opioid use and a decrease in emergence agitation 15 minutes postoperatively in the clonidine group.^23^ The meta-analysis on clonidine as an adjunct to peripheral nerve blocks in a pediatric population by Lundblad et al^51^ reported the addition of clonidine significantly prolonged the analgesic effect and reduced the number of patients that needed more than 2 doses of supplemental analgesics.

Concerning dexmedetomidine, Mostafa et al^6^ compared a group that received an SZMNB containing only bupivacaine to a group that received bupivacaine combined with dexmedetomidine in a pediatric population undergoing cleft palate surgery. They described statistically significant decreased pain scores at 8 hours postoperative and decreased need for postoperative analgesics in the dexmedetomidine group.^6^ However, the sedation score was significantly higher in the intervention group during the first postoperative hour.^6^ In addition, the meta-analysis by Yang et al^52^ showed a significantly decreased postoperative analgesic requirement and prolonged analgesic effect when dexmedetomidine was added as an adjunct to peripheral nerve blocks in pediatric populations.

Supplemental Table 2, Supplemental Digital Content 7, http://links.lww.com/SCS/G349 provides an overview of the recommended MNB administration technique.

## DISCUSSION

Survey results showed a large heterogeneity among the respondents in the used MNB techniques. A suprazygomatic approach with needle reorientation towards the ipsilateral commissure before incision was most frequently reported. The majority of respondents that used an MNB before the course did not use ultrasound-guided administration.

In the literature, various techniques are described for MNB administration. Since both intraoral techniques require identifying the second and third molars,^13,14,17^ which will often not yet be present in young children, these approaches are not suitable for this population. With regard to the transfacial approaches to the PPF, Jerman et al^53^ assessed both the feasibility and safety of suprazygomatic, anterior infrazygomatic, and posterior infrazygomatic approaches using virtual reality on 3-dimensional reconstructed CT angiography scans of 100 adult patients. They found the suprazygomatic approach to be feasible in 96.5% of the cases, and it proved safe in all feasible cases, meaning there was no risk of MA puncture.^53^ The posterior and anterior infrazygomatic approaches were both feasible in all cases and proved safe in 73.5% and 38% of the cases, respectively.^53^ The risk of passing the needle through the inferior orbital fissure was not considered.^53^ This is a possible complication when using a posterior infrazygomatic approach,^18^ but it is anatomically impossible when using a suprazygomatic or anterior infrazygomatic approach to the PPF.^47^ In general, the suprazygomatic technique is considered the safest and is subsequently the most frequently described approach to the PPF in clinical papers on MNB in pediatric populations.^2,4–6,11,20–23^

With regard to the SZMNB needle reorientation landmarks, the anterior aspect of the contralateral tragus is described to be the most favorable,^44,46,48^ which we also observed during dissection at the ICPMCA23. Nevertheless, the survey most frequently reported reorientation towards the ipsilateral commissure. Furthermore, the nasolabial fold^20^ and the philtrum^2,4–6,11,22,23^ are the most frequently described landmarks in clinical papers on SZMNB in children. This could be because the research group of Captier et al,^47^ who performed an anatomic study using CT scans of infants aged 1 week to 16 months and described the philtrum as the preferred reorientation landmark, also published multiple clinical studies.^2,11,22^

In all clinical studies, the SZMNB was performed before incision,^2,4–6,11,20–22^ and studies indicate that less intraoperative analgesia is needed when using this approach.^4,11^ Since children under the age of 3 years may be at risk of possible neurotoxicity caused by general anesthetics,^54^ the decreased need for intraoperative analgesia could be an advantage. Lidocaine, with or without epinephrine, could be added as local submucosal infiltration before incision to improve hemostasis.^5,23^ Furthermore, it will provide additional peri-operative analgesia for ∼ 1.5 to 2 hours.^55^ Since lidocaine is also an amino-amide class anesthetic, it adds to the maximum dosage regarding systemic toxicity when added to an SZMNB containing either ropivacaine or (levo)bupivacaine.^56^ However, lidocaine has a rather short half-life time, and the addition of adrenaline to lidocaine decreases systemic toxicity.^56^ Nevertheless, a postoperative SZMNB administration could be taken into consideration when adding local submucosal infiltration with lidocaine before incision since there is less risk of systemic toxicity when administering the SZMNB containing either ropivacaine or (levo)bupivacaine after lidocaine plasma concentration has become negligible. Furthermore, a postoperative administration may result in a longer optimal effect of the MNB during the direct postoperative period.

To our knowledge, no studies have been conducted comparing pre-operative to postoperative administration of the SZMNB in children undergoing cleft palate surgery with regard to peri-operative and postoperative narcotic usage. The decision to administer the MNB prior to or after incision is therefore most likely dependent on the specialist’s preference and training.

Due to the well-defined landmarks and the safety of the suprazygomatic approach, the SZMNB could be administered blindly.^5^ Nevertheless, ultrasound guidance should be used whenever available since it provides an added degree of safety given the proximity and anatomic variation of the MA within the infratemporal fossa and PPF, allowing for more accurate placement of the anesthetic.^5,22^

To avoid inadvertent vascular punctures or intravascular injections, color Doppler should be used during needle advancement or local anesthetics injection. Furthermore, we advise using an echogenic needle since, otherwise, the needle will not be visible if the angle to the transducer is more than 45 degrees. Unfortunately, the maxillary nerve will appear anisotrophic on ultrasound in both suprazygomatic and infrazygomatic techniques, probably since it is not hit perpendicular by the ultrasound beam. When using a suprazygomatic approach the needle tip will be visible out of plane.^22^ However, both the anterior and posterior infrazygomatic approaches result in an in-plane complete ultrasound visualization of the needle,^43^ and could therefore be favored during ultrasound-guided regional anesthesia. It is important to realize that the 2 bony landmarks visible on the ultrasound image correspond to the maxilla and coronoid process of the mandible^44^ as opposed to the greater wing of the sphenoid as suggested in some literature.^4,22,48^ This can be easily checked by moving the mandible with the transducer in place. The landmark will move when opening and closing the mouth, confirming it to be the coronoid process of the mandible (mobile landmark), in contrast to the sphenoid bone, which would be a non-mobile landmark.

When performing regional anesthesia in pediatric populations, amino-amide class local anesthetics are preferred due to the prolonged duration of action.^57^ Since a long-acting analgesic is preferred in an MNB, ropivacaine or (levo)bupivacaine are the most frequently described analgesics for MNB administration with regard to cleft surgery.^2,5,11,20–22^ In the survey, the reported concentration of 3% ropivacaine (n=1) is much higher than the maximum concentration of 1% available on the market. Therefore, the most plausible reason for this high reported concentration is a mistake by the survey respondent. Frequently reported concentrations of ropivacaine and (levo)bupivacaine in the literature are, respectively, 0.15 ml/kg 0.2% to 0.25%^2,5,11,22^ and 0.15 to 0.2 ml/kg 0.125% to 0.25%^4,6,20,23^ per side. These concentrations are lower than the dosing recommendations of the American and European Society for Regional Anesthesia and Pain Therapy (ASRA/ESRA) for peripheral nerve blocks of the upper and lower extremity of 0.5 to 1.5 ml/kg 0.2% ropivacaine or 0.25% bupivacaine.^55,57^ This is probably due to the MNB being placed in a small anatomic space close to the nerve which requires smaller volumes. Nevertheless, despite this dose being only 25% of the maximum dose, systemic toxicity can occur in case of intravascular injection of the local anesthetic. Therefore, awareness and ultrasound visualization of the variance and asymmetry of the MA course in the infratemporal fossa is of great importance. Furthermore, total maximum dose with regard to systemic toxicity should be taken into consideration when combining multiple anesthetics of the amino-amide class, such as ropivacaine, bupivacaine, and lidocaine.^56^

All clinical studies on the use of MNB in children used weight-dependent anesthesia dosages.^2–6,11,20–23^ Some studies, that also involved older children or adults, described maximum total injected volumes per side of 4^6,20^ or 5 ml.^3,23^ Both Mireault et al^44^ and Echaniz et al^48^ performed an anatomic study on embalmed cadaveric adult heads in which they injected methylene blue dye using an ultrasound-guided suprazygomatic approach. With an injected volume of 5 ml per side, Mireault et al^44^ found the methylene blue dye to have spread from the infratemporal fossa to the PPF and maxillary nerve. Echaniz et al^48^ injected half of the specimens with 5 ml per side and the other half with only 1 ml per side, described to be in accordance with the calculated adult volume of the PPF. They found the methylene blue dye in the high volume group not only in the PPF, but also in the infratemporal fossa, above the temporal muscle, and at the masseteric, temporal, and mandibular nerve branches. However, in the low volume group, the methylene blue dye had only sporadically spread sufficiently into the PPF. Therefore, they suggest a volume of slightly over 1 ml per side should allow for effective analgesia of the maxillary nerve.^48^ However, since the needle tip will most likely be positioned near the entrance of the PPF as opposed to within the PPF, analgesia of the maxillary nerve depends on the diffusion of the anesthetic from the infratemporal fossa towards the PPF. Since no complications from spread into adhering compartments have been described in the literature,^3,6,20,23^ we advise injecting a maximum total volume of 3 to 4 ml on each side.

The duration of action of long-acting local anesthetics is ∼4 to 12 hours, which does not cover the entire postoperative painful period of 24 to 72 hours associated with moderate or major surgery, such as cleft palate repair.^55^ Therefore, adjuvants have been added to single-injection peripheral nerve blocks to prolong the analgesic effect and allow for a smoother emergence from anesthesia.^55^

The addition of dexamethasone IV has been described to have a positive effect on intraoperative and postoperative opioid consumption and time to first oral intake in a pediatric population.^21^ Furthermore, both perineural and IV dexamethasone have proven to significantly increase the sensory block time of local anesthetics in adult populations.^50,58,59^ However, underlying mechanisms of analgesia prolongation are unclear,^58^ it is difficult to distinguish systemic from local effects of dexamethasone^49,50,58^ due to the high systemic absorption in case of a perineural administration,^59^ and there is lack of evidence in pediatric populations.^50^ This causes perineural dexamethasone not to meet the fundamental requirements of adjuvant drugs stated by the ASRA/ESRA^55^ and should, therefore, not be routinely added in children. Nevertheless, dexamethasone is frequently administered IV to reduce postoperative nausea, vomiting, and pain.^60^

Both clonidine and dexmedetomidine could be used as an adjunct to peripheral nerve blocks.^58^ However, regarding clonidine, it is still unclear which local anesthetics, anatomic blocks or doses of clonidine are optimal despite the substantial research that has been conducted.^49^ Furthermore, the addition of clonidine as an adjunct to peripheral nerve blocks with intermediate or long-acting local anesthetics increases the risk of hypotension.^61^ Due to the more favorable pharmacokinetic and pharmacodynamic properties and longer duration of sensory block time, dexmedetomidine could be favored over clonidine as an adjuvant to local anesthesia in peripheral nerve blocks.^58^ In addition, dexmedetomidine meets the requirements of the ASRA/ESRA for adjuvant drugs^55^ with meta-analysis supported evidence of the beneficial effects in both adult^49,58,59^ and pediatric populations,^52^ known mechanism of action, and it has a tolerable side effect profile. However, it only has a prolonging analgesic effect when added as a perineural adjunct and not when added IV.^59^ The literature review by Kirksey et al^49^ shows that there is vast evidence for dexmedetomidine to prolong the analgesic effect of peripheral nerve blocks by approximately 200 minutes at doses around 1 µg/kg in adults, mainly when added to ropivacaine or bupivacaine. Therefore, a maximum dose of 1 µg/kg dexmedetomidine could be added as a perineural adjunct to the MNB in children undergoing cleft palate surgery on a routine basis. However, the possible side effects, such as sedation, bradycardia, and hypotension, must be taken into consideration.^58^

A limitation of this study is the low response rate and missing data in the ICPMCA23 survey. The actual number of registrants that attended the course online, completely or partly, could not be determined with certainty. Therefore, we sent the survey to all course registrants. The low response rate could be explained by not all registrants attending the course. The missing data could be explained by the length of the survey, which may have led respondents to answer only the first few questions. The extensive number of questions could also have influenced the quality of the given answers. There were no questions on other aspects of cleft care or possible complications of MNB administration incorporated in the survey. This could also be considered a limitation of our study. Furthermore, the low response rate and the selection of participants of the congress, gives a bias towards practitioners interested in the block technique. Nevertheless, the survey shows a large heterogeneity in techniques, which proves the need for more consensus about the required technique of MNB administration.

Another limitation is the narrative nature of our literature review. However, our literature search is clearly defined and can be checked and repeated. Furthermore, the heterogeneity of studies on this subject makes a systematic review and meta-analysis difficult. Nevertheless, this study does provide a comprehensive overview of the current literature on the subjects of infratemporal fossa and PPF anatomy, MA variations, described techniques of MNB administration, commonly used anesthetics and adjuvants and the added value of ultrasound guidance supplemented with the expert opinion of the authors. This will, hopefully, lead to more awareness and homogeneity in MNB administration in the context of global cleft palate surgery.

## CONCLUSION

The MNB is not yet routinely used globally as part of cleft palate surgery-related pain management, and described techniques are heterogeneous throughout the literature and among the ICPMCA23 survey respondents.

When performing an MNB, the suprazygomatic approach is regarded as the safest. When using the suprazygomatic approach, reorientation of the needle towards the anterior aspect of the contralateral tragus will result in the needle tip positioned closest to the entrance of the PPF. Needle reorientation angles do not have to be adjusted to age, unlike needle depth. An echogenic needle should be used in combination with ultrasound guidance whenever available. A long-acting local anesthetic at a concentration of 0.2% to 0.25% 0.15 ml/kg with a maximum total injected volume of up to 4 ml per side is most widely used. A maximum dose of 1 µg/kg dexmedetomidine could be added as a perineural adjunct to the MNB to prolong the analgesic effect.

Further research is required comparing different techniques regarding efficacy and safety.

## Supplementary Material
