# Supplementary material for: The Maxillary Nerve Block in Cleft Palate Care: A Review of the Literature and Expert’s Opinion on the Preferred Technique of Administration
Source: J Craniofac Surg. 2024 Jun 11;35(5):1356–63. doi: 10.1097/SCS.0000000000010343 (PMC11198960; doi:10.1097/SCS.0000000000010343)
Supplement: Supplementary file 1 [file scs-35-1356-s001.docx]

# Supplemental appendix A

## International Cleft Palate Master Course Amsterdam 2023 survey

- 1. **I give permission to be contacted again for future research.**

1. Yes
2. No
   1. **In which country are you situated?**
   2. **In which city is your hospital located?**
   3. **What is your profession?**
   4. Plastic surgeon
   5. Oral and Maxillofacial surgeon
   6. Anesthesiologist
   7. ENT surgeon
      1. **Please specify your profession.**
   8. **How would you rate the streaming of the presentations on Thursday (were you able to follow everything)?**
   9. Excellent (no breaks in connectivity)
   10. Good (stable, 1-2 short breaks in connectivity)
   11. Moderate (4-5 short breaks in connectivity)
   12. Bad (unstable, >5 short and/or 1-2 long breaks in connectivity)
   13. Very bad (very unstable, >5 short and/or >2 long breaks in connectivity)
   14. **How would you rate the quality and content of the presentations on Thursday, did you find the information useful?**
   15. Excellent
   16. Good
   17. Moderate
   18. Bad
   19. Very bad
   20. **How would you rate the streaming of the presentations on Friday (were you able to follow everything)?**
3. Excellent (no breaks in connectivity)
4. Good (stable, 1-2 short breaks in connectivity)
5. Moderate (4-5 short breaks in connectivity)
6. Bad (unstable, >5 short and/or 1-2 long breaks in connectivity)
7. Very bad (very unstable, >5 short and/or >2 long breaks in connectivity)
   1. **How would you rate the quality and content of the presentations on Friday, did you find the information useful?**
8. Excellent
9. Good
10. Moderate
11. Bad
12. Very bad
    1. **How would you rate the streaming of the live dissection on Friday (were you able to follow everything)?**
13. Excellent (no breaks in connectivity)
14. Good (stable, 1-2 short breaks in connectivity)
15. Moderate (4-5 short breaks in connectivity)
16. Bad (unstable, >5 short and/or 1-2 long breaks in connectivity)
17. Very bad (very unstable, >5 short and/or >2 long breaks in connectivity)
    1. **How would you rate the quality and content of the live dissection on Friday, did you find the information useful?**
18. Excellent
19. Good
20. Moderate
21. Bad
22. Very bad
    1. **Did you miss any information during the presentations or dissection (i.e. information you wish had been given)**
23. No
24. Yes
    - 1. **Please specify what you missed.**
    1. **Did you use any type of regional anesthesia at the maxillary nerve during or after cleft palate surgery in your practice prior to the course?**
25. Yes
26. No
    - 1. **What regional anesthetic do you use?**
27. Bupivacaine
28. Ropivacaine
29. Lidocaine
30. Other
    - - 1. **Please specify the concentration of anesthetic you use.**
      1. **Do you use an adjuvant to the regional anesthetic?**
31. No
32. Yes
    - - 1. **What adjuvant do you use?**
33. Clonidine
34. Dexmedetomidine
35. Dexamethasone
36. Other
    - - - 1. **Please specify the concentration of adjuvant you use**
        1. **Please specify the concentration of adjuvant you use**
        2. **How do you administer the adjuvant**
37. IV
38. Within the block
39. Other
    - - - 1. **Please specify how you administer the adjuvant**
      1. **Do you use ultrasound guidance during administration of the nerve block?**
40. Yes
41. No
42. Sometimes
    - - 1. **If you sometimes use ultrasound guidance, please specify when**
      1. **What is your preferred way of administration?**
43. Suprazygomatic
44. Infrazygomatic
45. Intraoral
46. Other

- - - 1. **Please specify your preferred way of administration.**
      2. **To which landmark do you aim your needle after changing the angle?**

1. Ipsilateral commisure
2. Philtrum
3. Contralateral tragus
4. Other
   - - - 1. **Please specify to what landmark you aim you needle after changing the angle.**
     1. **Do you use local infiltration of anesthetics in the palate in addition to the nerve block?**
5. Yes
6. No
7. Sometimes
   - 1. **At what moment do you administer the nerve block?**
8. Prior to incision
9. Post-operative
10. Other
    - - 1. **Please specify when you administer the nerve block**
      1. **Do you intend to either start using the block or alter your technique of administration due to the course?**
11. Yes
12. No
13. Maybe
    - - 1. **What regional anesthetic will you use?**
14. Bupivacaine
15. Ropivacaine
16. Lidocaine
17. Other
    - - - 1. **Please specify what regional anesthetic you will use.**
        1. **Please specify the amount and concentration of regional anesthetics you will use**
        2. **Will you use an adjuvant to the local anesthetic?**
18. Yes
19. No
    - - - 1. **What adjuvant will you use?**
20. Clonidine
21. Dexmedetomidine
22. Dexamethasone
23. Other

**Please specify what adjuvant you will use.**

- - - - 1. **Please specify the concentration of adjuvant you will use.**
        2. **How will you administer the adjuvant**

1. IV
2. Within the block
3. Other

**Please specify how you will administer the adjuvant.**

- - - 1. **Will you use ultrasound guidance during administration of the nerve block?**

1. Yes
2. No
3. Sometimes
   - - 1. **What technique of administration will you use?**
4. Suprazygomatic
5. Infrazygomatic
6. Intraoral
7. Other
   - - - 1. **Please specify what technique you will use.**
         2. **To which landmark will you aim you needle after changing the angle?**
8. Ipsilateral commisure
9. Philtrum
10. Contralateral tragus
11. Other

**Please specify to which landmark you will aim you needle after changing the angle.**

- 1. **What are the three most important/interesting things you have learned during the course?**
  2. **Do you have any suggestions for improvement of the course?**
