# Supplementary material for: The Maxillary Nerve Block in Cleft Palate Care: A Review of the Literature and Expert’s Opinion on the Preferred Technique of Administration
Source: J Craniofac Surg. 2024 Jun 11;35(5):1356–63. doi: 10.1097/SCS.0000000000010343 (PMC11198960; doi:10.1097/SCS.0000000000010343)
Supplement: Supplementary file 2 [file scs-35-1356-s002.docx]

# Supplemental table 1

| Characteristic | Number of respondents (%) |
| --- | --- |
| Discipline |  |
| Plastic surgeon | 23 (29.5) |
| Oral & maxillofacial surgeon | 15 (19.2) |
| Anesthesiologist | 25 (32.1) |
| ENT surgeon | 1 (1.2) |
| Other (pediatric/general surgeon) | 14 (17.9) |
| LOCATION |  |
| eUROPE | 11 (14.1) |
| aFRICA | 23 (29.5) |
| aSIA | 30 (38.5) |
| SOUTH AMERICA | 11 (14.1) |
| oCEANIA | 1 (1.3) |
| Unknown | 2 (2.6) |
