# Supplementary material for: The Maxillary Nerve Block in Cleft Palate Care: A Review of the Literature and Expert’s Opinion on the Preferred Technique of Administration
Source: J Craniofac Surg. 2024 Jun 11;35(5):1356–63. doi: 10.1097/SCS.0000000000010343 (PMC11198960; doi:10.1097/SCS.0000000000010343)
Supplement: Supplementary file 3 [file scs-35-1356-s003.docx]

# Supplemental appendix B

## Flowchart of study inclusion maxillary artery anatomy

**PubMed search: 233 articles**

(“maxillary artery”[Mesh]” OR “maxillary arter*”[tiab]) AND anatomy

7 articles excluded

- 5 articles not describing MA course first two segments
- 1 case reports
- 1 no full text

3 relevant articles found through reference screening

10 articles included

14 articles

Full text screening

219 articles excluded

Title & abstract screening
