# Supplementary material for: The Maxillary Nerve Block in Cleft Palate Care: A Review of the Literature and Expert’s Opinion on the Preferred Technique of Administration
Source: J Craniofac Surg. 2024 Jun 11;35(5):1356–63. doi: 10.1097/SCS.0000000000010343 (PMC11198960; doi:10.1097/SCS.0000000000010343)
Supplement: Supplementary file 4 [file scs-35-1356-s004.docx]

# Supplemental appendix C

## Flowchart of study inclusion MNB in pediatric population

**PubMed search: 54 articles**

("Maxillary Nerve"[Mesh] OR ("maxillary”[tiab] AND “nerve*"[tiab])) AND (“Nerve block”[Mesh] OR (“nerve*”[tiab] AND “block*”[tiab])) AND ("Child"[Mesh] OR "child*"[tiab] OR "Infant"[Mesh] OR "infant*"[tiab])

37 articles excluded

- 27 not relevant
- 6 not English
- 2 no full text available
- 1 letter to the editor
- 1 case report

Title & abstract screening

4 articles excluded

- 1 poor quality
- 2 not relevant
- 1 review; original articles included

17 articles

Full text screening

7 relevant articles found through reference screening

20 articles included
