# Supplementary material for: The Maxillary Nerve Block in Cleft Palate Care: A Review of the Literature and Expert’s Opinion on the Preferred Technique of Administration
Source: J Craniofac Surg. 2024 Jun 11;35(5):1356–63. doi: 10.1097/SCS.0000000000010343 (PMC11198960; doi:10.1097/SCS.0000000000010343)
Supplement: Supplementary file 5 [file scs-35-1356-s005.docx]

# Supplemental appendix D: Critical appraisal

## Randomized controlled trials

| **Author + year of publication** | **1** | **2** | **3** | **4** | **5** | **6** | **7** | **8** | **9** | **10** | **11** | **12** | **13** | **Overall appraisal** |
| --- | --- | --- | --- | --- | --- | --- | --- | --- | --- | --- | --- | --- | --- | --- |
| Abu Elyazed et al. 2018 * | Yes | Yes | Yes | Yes | Unclear | Yes | Unclear | Yes | Yes | Yes | Yes | Yes | Unclear | Moderate |
| Chiono et al. 2014 | Yes | Yes | Yes | Yes | Yes | Yes | Yes | Yes | Yes | Yes | Yes | Yes | Yes | Good |
| Echaniz Barbero et al. 2018 | Yes | Yes | Yes | Yes | No | Yes | Yes | Yes | Yes | No | Yes | Yes | Yes | Good |
| Echaniz Barbero et al. 2020 * | Yes | Yes | Yes | Yes | Yes | Yes | Yes | Yes | Yes | Yes | Yes | Yes | Yes | Good |
| Echaniz Barbero et al. 2021 | Yes | Yes | Yes | Yes | Unclear | Yes | Yes | Yes | Yes | No | Yes | Yes | Yes | Good |
| Mostafa et al. 2018 | Yes | Yes | Yes | Yes | Yes | Yes | Yes | Yes | Yes | Yes | Yes | Yes | Yes | Good |
| Mostafa et al. 2020 | Yes | Yes | Yes | Yes | Yes | Yes | Yes | Yes | Yes | No | Yes | Yes | Yes | Good |

## Analytical cross sectional studies

| **Author + year of publication** | **1** | **2** | **3** | **4** | **5** | **6** | **7** | **8** | **Overall appraisal** |
| --- | --- | --- | --- | --- | --- | --- | --- | --- | --- |
| Captier et al. 2009 | Yes | Yes | N.A. | Yes | Yes | Yes | Yes | Yes | Good |
| Malamed et al. 1983 * | Yes | Yes | N.A. | Yes | Yes | Yes | Yes | Yes | Good |
| Marston et al. 2017 | Yes | Yes | N.A. | Yes | Yes | Yes | Yes | Yes | Good |
| Mireault et al. 2021 | Yes | Yes | N.A. | Yes | Yes | Yes | Yes | N.A. | Good |
| Prigge et al. 2014 | Yes | Yes | N.A. | Yes | No | No | Yes | Yes | Moderate |
| Singh et al. 2001 * | Yes | Yes | N.A. | Yes | No | No | Yes | Yes | Moderate |
| Sola et al. 2012 | Yes | Yes | Yes | Yes | No | No | Yes | Yes | Moderate |

## Cohort studies

| **Author + year of publication** | **1** | **2** | **3** | **4** | **5** | **6** | **7** | **8** | **9** | **10** | **11** | **Overall appraisal** |
| --- | --- | --- | --- | --- | --- | --- | --- | --- | --- | --- | --- | --- |
| Cawthorn et al. 2022 | Yes | Yes | Yes | Yes | Yes | Yes | Yes | Yes | Unclear | Unclear | Yes | Good |
| Esfahanian et al. 2022 | Yes | Yes | Yes | Yes | Yes | Yes | Yes | Yes | Yes | No | Yes | Good |
| Mesnil et al. 2010 | Unclear | Yes | Yes | No | No | Yes | Yes | Yes | Unclear | No | No | Moderate |
| Stechison et al. 1994 * | N.A. | N.A. | N.A. | No | No | Yes | No | Yes | Yes | N.A. | N.A. | Poor |
| Sved et al. 1992 * | N.A. | N.A. | Yes | No | No | Yes | Yes | Yes | No | No | Yes | Moderate |

## Textual evidence: expert opinion

| **Author + year of publication** | **1** | **2** | **3** | **4** | **5** | **6** | **Overall appraisal** |
| --- | --- | --- | --- | --- | --- | --- | --- |
| Mercuri et al. 1979 * | Yes | Yes | Yes | Yes | No | No | Moderate/poor |
