# Supplementary material for: The Maxillary Nerve Block in Cleft Palate Care: A Review of the Literature and Expert’s Opinion on the Preferred Technique of Administration
Source: J Craniofac Surg. 2024 Jun 11;35(5):1356–63. doi: 10.1097/SCS.0000000000010343 (PMC11198960; doi:10.1097/SCS.0000000000010343)
Supplement: Supplementary file 6 [file scs-35-1356-s006.docx]

# Supplemental appendix E

## Study characteristics and extracted data

| **Author + year of publication** | **Study** | **Population** | **MNB technique** | **Moment of administration** | **Ultrasound guidance** | **Needle** | **Needle depth** | **Anesthetic** | **Adjuvant** |
| --- | --- | --- | --- | --- | --- | --- | --- | --- | --- |
| Abu Elyazed et al. 2018 * | SZMNB vs palatal block vs no block | Children age 3mo-2y n=90 | Suprazygomatic – not described | Prior to incision | Yes | 25G 50mm | 35-45mm | Bupivacaine 0.15ml/kg 0.25% | - |
| Captier et al. 2009 | Anatomical study | Children (3D CT) age 0-16 mo n=55 | Suprazygomatic - philtrum | - | - | - | - | - | - |
| Cawthorn et al. 2022 | SZMNB as part of new perioperative clinical care pathway vs retrospective cohort. | Children age 9-21 mo N=80 | Suprazygomatic - philtrum | Prior to incision | Yes | 24G 40mm | 30-35mm | Ropivacaine 0.15ml/kg 0.25% | - |
| Chiono et al. 2014 | SZMNB ropivacaine vs saline | Children age 1-29 mo n=57 | Suprazygomatic – nasolabial fold | Prior to incision | Yes | 25G 50mm | 35-45mm | Ropivacaine 0.15ml/kg 0.2% | - |
| Echaniz Barbero et al. 2018 | SZMNB vs infra-orbital & palatine nerve blocks | Children age 1-18y n=96 & adults n=18 | Suprazygomatic – philtrum  Also pre incision LA palate with lignocaine+epinephrine hemostasis | Prior to incision | No | 27G 38mm | 35-38mm | Bupivacaine 0.15ml/kg 0.25%  Max volume 5ml per side | Dexamethasone IV 0.2-0.3 mg/kg |
| Echaniz Barbero et al. 2020 * | Anatomical study | Adults (cadaver) n=4 | Suprazygomatic – contralateral tragus | - | Yes | 22G 90mm | 37mm | (Methylene blue dye 1 or 5 ml) | - |
| Echaniz Barbero et al. 2021 | SZMNB bupivacaine vs bupivacaine + clonidine | Children  Median age 5 and 7 yo n=119 | Suprazygomatic – philtrum | Prior to incision | No | 27G 38mm | 35-38mm | Bupivacaine 0.15ml/kg 0.25%  Max volume 5ml per side | 1 group clonidine 3mg/kg max 150mg |
| Esfahanian et al. 2022 | SZMNB vs historical cohort without SZMNB | Children age >6 mo n=102 | Suprazygomatic – not described | Prior to incision | Not described | Not described | Not described | Ropivacaine 1mg/kg/side | Dexamethasone IV 1x intraoperatively 0.5mg/kg, every 8 hours during 24h postoperative 0.25mg/kg |
| Malamed et al. 1983 * | Anatomical study | Adult skulls n=158 | Intraoral | - | - | - | - | - | - |
| Marston et al. 2017 | Anatomical study | Children (CT) age 0-18 years n=90 | Suprazygomatic - philtrum | - | - | - | - | - | - |
| Mercuri et al. 1979 * | Narrative review on anatomy & technique | - | Intraoral | - | - | - | - | - | - |
| Mesnil et al. 2010 | SZMNB effectiveness & complications. Analgesic consumption vs retrospective cohort. | Children age 3-12 mo n=33 | Suprazygomatic - philtrum | Prior to incision | No | 25G 50mm | 35-45mm | Ropivacaine 0.15ml/kg 0.2% | - |
| Mireault et al. 2021 | Anatomical study | Adult (cadaver) n=2 | Suprazygomatic - philtrum | - | Yes |  | 40mm | (methylene blue dye 5ml) | - |
| Mostafa et al. 2018 | SZMNB bupivacaine vs levobupivacaine | Children age 1-10 years n=60 | Suprazygomatic – nasolabial fold | Prior to incision | No? | 25G 50mm | 35-45mm | Levobupivacaine vs bupivacaine 0.15ml/kg 0.2%  Max volume 4ml per side | - |
| Mostafa et al. 2020 | SZMNB bupivacaine vs bupivacaine + dexmedetomidine | Children age 1-5 yo n=80 | Suprazygomatic – not described | Prior to incision | No | 25G 50mm | 35-45mm | Bupivacaine 0.2ml/kg 0.125%  Max volume 4ml per side | One group dexmedetomidine 0.5 µg/kg |
| Prigge et al. 2014 | Anatomical study | Children (cadaveric n=40 + CT) | Suprazygomatic - contralateral tragus | - | - | - | 20 mm neonates  30mm infants <1yo | - | - |
| Singh et al. 2001 * | Anatomical study | Adults n=75 + adult skulls n=120 | Anterior infrazygomatic | - | No | 100mm | 44-45mm | Not described | - |
| Sola et al. 2012 | SZMNB feasibility, pain scores, side effects & LA spread | Children age 6-62 mo n=25 | Suprazygomatic - philtrum | Prior to incision | Yes | 25G 50mm | 35-45mm | Ropivacaine 0.15ml/kg 0.2% | - |
| Stechison et al. 1994 * | Anatomical study | Adults n=4 | Anterior infrazygomatic | - | CT | 22G 89mm | Not described | Bupivacaine 0.5% 1.5-2ml | - |
| Sved et al. 1992 * | Complications of intraoral MNB | Children & adults 9-84yo n=101 | Intraoral | - | - | - | - | - | - |

Characteristics and extracted data of articles included on the subject of MNB in pediatric populations.

* Articles identified through reference screening

| **Author + year of publication** | **Population** | **Study** | **MA course in relation to LPM** | |  | **Asymmetry** |
| --- | --- | --- | --- | --- | --- | --- |
|  |  |  | **Medial (deep)** | **Lateral (superficial)** | **Intermediate** |  |
| Alvernia et al. 2017 | Caucasian | Anatomical study  6 adult cadaveric heads (12 sides) + 20 CT angiograms | 33% Cadaver  52.5% CTA | 66% Cadaver  47.5% CTA | - | - |
| Gulses et al. 2012 | Caucasian | Anatomical study  CT angiogram of 209 patients | 31.6% | 68.4% | - | 21.1% |
| Hussain et al. 2008 * | Caucasian | Anatomical study  44 adult cadaveric heads | 32% | 68% | - | - |
| Hwang et al. 2014 | Asian | Anatomical study  CT scans of 100 patients (200 sides) | 18% | 82% | - | 12% |
| Lovasz et al. 2023 | Caucasian | Anatomical study  CT scans of 50 adult patients (100 sides) | 37% | 56% | 7% | 18% |
| Maeda et al. 2012 | Asian | Anatomical study & review  104 adult cadaveric heads (208 sides) | 8.2%  Results review  38% Caucasians  7.3% Mongoloids | 90.4%  Results review  61.6% Caucasians  92.7% Mongoloids | 1.4% | 12.5% |
| Otake et al. 2011 | Asian | Anatomical study  15 adult cadaveric heads (28 sides) | 3.6% | 96.4% | 0.5-1.9% | - |
| Ottone et al. 2021 | Asian and Caucasian | Systematic review | 6.25-52.5% Caucasians  3-18% Asians | 47.5-93.8% Caucasians  82-87% Asians | 1.4-2.3% | 0-21% |
| Pretterklieber et al. 1991 * | Caucasian | Anatomical study  102 adult cadaveric heads | 45-47% | 53-55% | - | - |
| Uysal et al. 2011 * | Turkish | Anatomical study  7 adult cadaveric heads (14 sides) | 42.9% | 57.1% | - | - |

Characteristics and extracted data of articles included on the subject of maxillary artery anatomy.

* Articles identified through reference screening
