# Supplementary material for: The Maxillary Nerve Block in Cleft Palate Care: A Review of the Literature and Expert’s Opinion on the Preferred Technique of Administration
Source: J Craniofac Surg. 2024 Jun 11;35(5):1356–63. doi: 10.1097/SCS.0000000000010343 (PMC11198960; doi:10.1097/SCS.0000000000010343)
Supplement: Supplementary file 7 [file scs-35-1356-s007.docx]

# Supplemental table 2

| Ultrasound guidance | Yes |
| --- | --- |
| Needle type | Echogenic needle |
| Needle size | 24-27G 38-50mm |
| Approach | Suprazygomatic |
| Moment of administration | Prior to incision |
| Needle insertion | At the frontozygomatic angle perpendicular to the skin until the greater wing of the sphenoid bone is reached |
| Reorientation landmark | Anterior aspect of contralateral tragus; no adjustment to age |
| Needle depth | Neonates: 20 mm   - 1. years old: 30-38.55mm   18 years old: 47.11mm |
| Anesthetic | Ropivacaine or (levo)bupivacaine |
| Dosage of anesthetic | 0.2 to 0.25% 0.15ml/kg |
| Volume of anesthetic | Maximum 4ml per side |
| Adjuvant | Dexmedetomidine |
| Concentration of adjuvant | 1microg/kg |
| Administration of adjuvant | Perineural |
